# Ginsenoside Rg3 (Shenyi Capsule) Combined with Chemotherapy for Digestive System Cancer in China: A Meta-Analysis and Systematic Review

**DOI:** 10.1155/2019/2417418

**Published:** 2019-12-17

**Authors:** Linlin Pan, Tingting Zhang, Haiyang Sun, Guirong Liu

**Affiliations:** ^1^Department of Chinese Medicine Literature and Culture, Shandong University of Traditional Chinese Medicine, Jinan 250355, China; ^2^Department of First Clinical Medical College, Shandong University of Traditional Chinese Medicine, Jinan 250355, China; ^3^Department of Traditional Chinese Medicine, Shandong University of Traditional Chinese Medicine, Jinan 250355, China

## Abstract

**Objective:**

In China, ginsenoside Rg3 is often used in combination with chemotherapy to treat digestive system cancer. We here performed a meta-analysis and systematic review to provide a much needed high-quality evaluation of the efficacy and safety of ginsenoside Rg3 combined with chemotherapy in these cancers.

**Materials and Methods:**

The PubMed, EMBASE, Cochrane Library, China National Knowledge Infrastructure (CNKI), Wanfang, and Weipu (VIP) databases were searched. All randomized controlled trials (RCTs) concerning ginsenoside Rg3 combined with chemotherapy for digestive system cancer were selected. Dichotomous data were expressed as odds ratios (ORs) with 95% confidence intervals (CI). The methodological quality of the included studies was evaluated according to the Cochrane evidence-based medicine system, and the statistical analyses were performed with Review Manager 5.3 and STATA 12.0 software. In addition, the Grades of Recommendation Assessment, Development and Evaluation (GRADE) approach was used to rate the quality of the evidence. Trial sequential analysis (TSA) was used to evaluate information size and treatment benefits.

**Results:**

A total of 18 trials comprising 1531 patients were included in this study. The results revealed that the trials were of sufficient standard to draw reliable conclusions that ginsenoside Rg3 combined with chemotherapy could improve the objective response rate (ORR; OR 2.17, 95% CI 1.72–2.73), disease control rate (DCR; OR 2.83, 95% CI 2.02–3.96), 1-year survival rate (SR; OR = 2.33, 95% CI = 1.24–4.37), Karnofsky Performance Scale (KPS; OR 2.67, 95% CI 1.76–4.03), gastrointestinal dysfunction (OR 0.44, 95% CI 0.31–0.61), and the decline of leucocyte count (OR 0.28, 95% CI 0.21–0.38).

**Conclusion:**

Ginsenoside Rg3 combined with chemotherapy can improve the clinical efficacy and alleviate treatment-induced side effects for digestive system cancer.

## 1. Introduction

Digestive system cancer is the most common and lethal cancer in the world, mainly including gastric cancer (GC), colorectal cancer (CRC), esophageal cancer (EC), liver cancer (LC), and pancreatic cancer (PC). At present, the changes in dietary patterns are considered to be the main causes of digestive system cancers [1]. Patients with digestive system cancer are often diagnosed at a middle or later stage, at which point radiotherapy or chemoradiotherapy is often used [2]. Even after treatment, overall survival and quality of life remain poor in advanced stages [3, 4]. Therefore, improving the early diagnosis and treatment of digestive system cancer is urgently needed.

Shenyi capsule (National Drug Administration standard number: Z20030044), an important Chinese anticancer drug, is composed of the ginseng root extract component, ginsenoside Rg3. Previous studies have found that ginsenoside Rg3 inhibits tumor growth through suppression of pathways linked to oncogenesis, including cell survival, proliferation, invasion, and angiogenesis [5, 6]. For example, angiogenesis can promote growth and metastasis of cancer [7] and is mediated in large part by vascular endothelial growth factor (VEGF) [8]; ginsenoside Rg3 can decrease the expression of VEGF, and its antitumor effects may be mediated through suppression of ERK and Akt signaling [9]. In addition, ginsenoside Rg3 combined with chemotherapy prolongs the survival time of patients and reduces adverse chemotherapy-induced reactions [10]. Therefore, Shenyi capsule is widely used as an adjuvant to chemotherapy in the treatment of various cancers in China. To better evaluate the clinical efficacy and safety of ginsenoside Rg3 combined with chemotherapy for digestive system cancers, we conducted the first systematic review and meta-analysis based on randomized controlled trials (RCTs).

## 2. Materials and Methods

This article was based on the Preferred Reporting Items for Systematic Reviews and Meta-analyses guidelines (PRISMA guidelines), and the materials were published studies.

### 2.1. Search Strategies

Studies were retrieved from the PubMed, EMBASE, Cochrane Library, China National Knowledge Infrastructure (CNKI), Wanfang, and Weipu (VIP) databases. All searches were implemented using MeSH and free words. The search period lasted from the established time to July 28, 2019. All the studies were searched regardless of their publication type and language. Two authors (Linlin Pan and Tingting Zhang) independently searched all the related studies in English and Chinese databases using the following search strategies:

For Chinese databases, the following terms were used in combined ways: [Renshen zaodai Rg3 OR Shenyi jiaonang] AND [Hualiao ] AND [Weiai OR Chang ai OR Shidaoai OR Ganai OR Yixianai ]. For English databases, the following terms were used in combined ways: [ Ginsenoside Rg3 OR 20s-ginsenoside rg3 OR Beta-D-glucopyranoside OR 20(R)-ginsenoside Rg(3) OR Ginsenoside 20-rg3 OR Shenyi capsule] AND [Chemotherapy OR Chemotherapies] AND [(Gastric Neoplasms OR Stomach Neoplasms OR Stomach Cancers OR Gastric Cancers OR Stomach tumors OR Gastric tumors OR Gastric Carcinomas OR Stomach Carcinomas) OR (Colorectal Neoplasms OR Colorectal Tumors OR Colorectal cancers OR Colorectal Carcinomas) OR (Esophageal Neoplasms OR Esophageal Cancers OR Esophageal Carcinomas OR Esophageal Tumors OR Esophagus Neoplasms OR Esophagus Cancers OR Esophagus Carcinomas OR Esophagus Tumors) OR (Liver Neoplasms OR Liver Cancers OR Liver tumors OR Liver Carcinomas OR Hepatocellular carcinoma) OR (Pancreatic Neoplasms OR Pancreatic Cancers OR Pancreatic tumors OR Pancreatic Carcinomas)]

### 2.2. Inclusion and Exclusion Criteria

#### 2.2.1. Inclusion Criteria

The inclusion criterias were as follows: (a) interventions: the control group treated with chemotherapy or chemotherapy plus placebo, and the experimental group was treated with ginsenoside Rg3 on the basis of the control group; (b) types of studies: RCTs; (c) participants: patients who were pathologically diagnosed with GC, CRC, EC, LC, or PC; (d) outcomes: objective response rate (ORR), disease control rate (DCR), survival rate (SR), Karnofsky Performance Scale (KPS), and side effects (the decline of leucocyte count, gastrointestinal dysfunction, and the hepatic and renal dysfunction).

#### 2.2.2. Exclusion Criteria

The exclusion criterias were as follows: (a) nonclinical experimental studies (such as reviews, protocols, and animal or cell research studies); (b) patients with other malignancies or serious medical diseases; (c) patients treated with other antitumor Traditional Chinese Medicine (TCM) drugs or therapies (such as acupuncture, massage, moxibustion, and Taiji).

### 2.3. Literature Selection and Data Extraction

The trials were selected from the relevant literature; two independent authors (Linlin Pan and Tingting Zhang) evaluated each title, abstract, and full-texts and then selected the relevant studies according to the inclusion criteria, and the discrepancies were settled through a consensus discussion. The following information was extracted from the included studies: the name of the first author, year of publication, type of cancer, sample size, sex, age, interventions, follow-up time, outcome measures, and criteria for efficacy.

### 2.4. Risk of Bias in Individual Trials

The methodological quality of each RCT was independently assessed by two authors (Linlin Pan and Tingting Zhang) using the Cochrane Risk of Bias tool [11]. Disagreements were discussed and resolved by the third reviewer (Guirong Liu). The following criteria were assessed: random sequence generation, allocation concealment, blinding of participants and personnel, blinding of outcome assessment, incomplete outcome data, selective reporting, and other biases. The risk of bias was classified as “high,” “unclear,” or “low.”

### 2.5. Data Synthesis and Analysis

The Review Manager (RevMan) Version 5.3 software was used to perform statistical analyses. The risk ratio (OR) with the correspondent 95% confidence intervals (CI) was used to pool the total effectiveness rates of dichotomous data, and *P* < 0.05 was considered statistically significant. The Chi^2^ and *I*^2^ tests were used to evaluate the heterogeneity, and the exhibited heterogeneity was *P* < 0.10 or *I*^2^ >50%. The fixed-effect model (FEM) was used for the merging of homogeneity data, and the random effects model (REM) was used for heterogeneous data merging. Sensitivity analysis was assessed by reanalyzing the data using various statistical methods. Publication bias was evaluated by visual assessment of the asymmetry of the funnel plots and Egger's test (STATA 12.0), where *P* < 0.05 indicated potential bias.

The GRADE approach [12] was used to rate the quality of the evidence. The quality of the evidence was classified as “high,” “moderate,” “low,” and “very low” level; downgraded according to the following aspects: limitation of the study design, inconsistency, indirectness, imprecision, and publication bias; and upgraded according to the following aspects: large effect, plausible confounding would change the effect, and dose-response gradient.

TSA software (version 0.9.5.10 Beta) was used to calculate the required information size (RIS) for meta-analysis and evaluate treatment benefits based on the sample sizes. The risk of type I error was set at 5% with a power of 80%, the variance was calculated from the data obtained from the included trials, and the relative risk reduction was set at 20% [13]. When cumulative Z-curves crossed sequential monitoring boundaries, a sufficient level of evidence is obtained for the intervention. When Z-curves did not cross the boundaries, the conclusions for the intervention are not justified [14].

## 3. Results

### 3.1. Overview of the Literature Search

**Figure 1:**
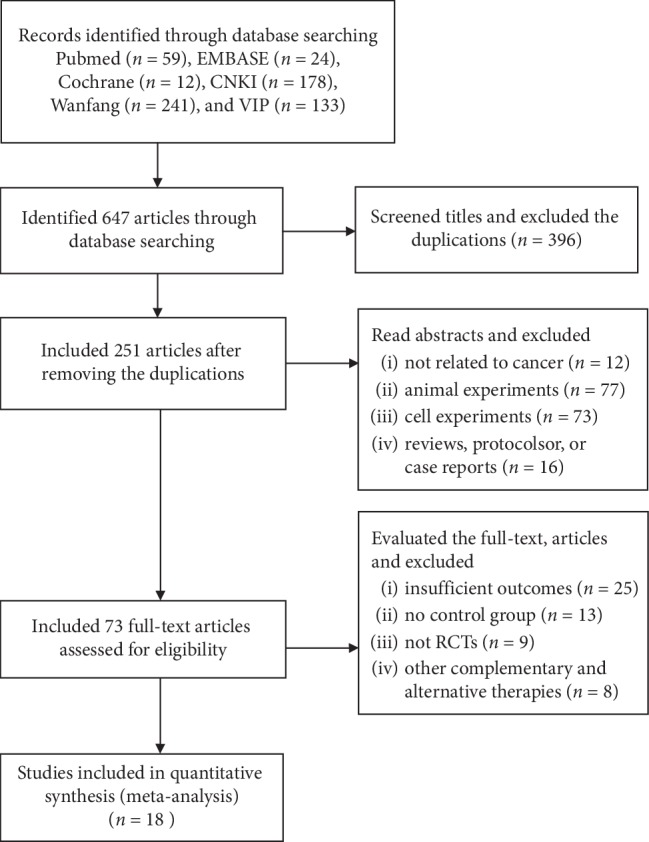
Flow diagram of the literature search process.

A total of 647 studies were identified during the initial database search. We used EndNote to exclude 396 duplications. We then read the abstracts and excluded animal experiment studies (*n* = 77), cell experiment studies (*n* = 73), reviews (*n* = 8), protocols (*n* = 6), case reports (*n* = 2), and experimental studies not related to cancer (*n* = 12). After reading the full-text articles, another 55 studies were excluded due to insufficient outcomes (*n* = 25), no control group (*n* = 13), not RCTs (*n* = 9) or using other complementary and alternative therapies (*n* = 8). Ultimately, 18 studies were included in the analysis (Figure 1).

#### 3.1.1. Study Characteristics

Eighteen studies published in 2009–2018 with a total of 1531 patients were included in this analysis, with 776 patients in experimental groups and 755 in control groups. There were four GC trials with 469 patients, five CRC trials with 384 patients, three EC trials with 337 patients, five LC trials with 273 patients, and one PC trial with 68 patients. The experimental groups underwent treatment with ginsenoside Rg3 (20 mg each time, twice daily) on the basis of chemotherapy, while the control groups underwent chemotherapy alone or chemotherapy plus placebo. The characteristics of the studies are shown in Table 1.

#### 3.1.2. Quality Assessment

**Figure 2:**
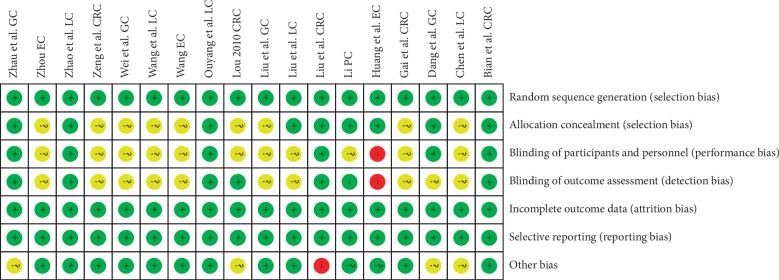
Risk of bias summary: review of authors' judgments about each risk of bias. Green refers to low risk of bias; yellow refers to unclear risk of bias; red refers to high risk of bias.

As shown in Figure 2, all included trials were RCTs and did not have incomplete outcome data (attrition bias) or selective reporting (reporting bias). Nine trials described the allocation concealment method, and others were unclear. Six trials used blind method for participants and personnel, one trial (Huang et al. EC) used open method, and others were unclear. Seven trials had a low risk in blinding of outcome assessment, one trial (Huang et al. EC) was an open experiment study with a high risk in detection bias, and others were unclear. As for other bias, one trial (Liu et al. CRC) was sponsored by a pharmaceutical company that produces Shenyi capsule and thus may have a high risk in its results, and four trials were unclear about the patients' sex and age. As shown in Table 2, the GRADE assessment revealed that the quality of the evidence for ORR, DCR, SR, KPS, leucocyte count, and gastrointestinal dysfunction was moderate, while the quality for hepatic and renal dysfunction was low.

### 3.2. Primary Outcomes

#### 3.2.1. ORR

**Figure 3:**
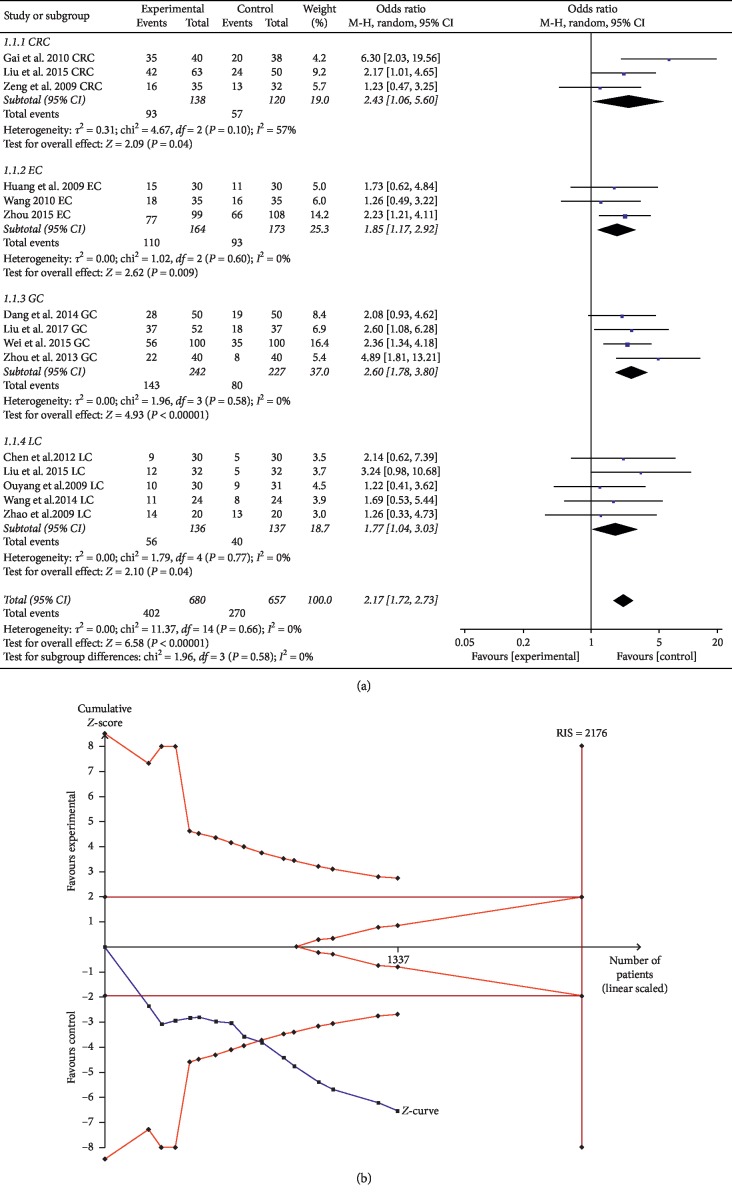
(a) Forest plot of ORR in the experimental group and control group. (b) Trial sequential analysis of ORR.

The ORR of ginsenoside Rg3 combined with chemotherapy was evaluated in a total of 15 trials comprising 1337 patients. Subgroups were divided according to different types of cancer: three CRC, three EC, four GC, and five LC. The ORR in the experimental group was significantly higher than that in the control group among the digestive system cancers included in this study; for CRC, OR = 2.43, 95% CI: 1.06–5.60, and *P*=0.04 in the *Z* test; for EC, OR = 1.85, 95% CI: 1.17–2.92, and *P*=0.009 in the *Z* test; for GC, OR = 2.60, 95% CI: 1.78–3.80, and *P* < 0.00001 in the *Z* test; and for LC, OR = 1.77, 95% CI: 1.04–3.03, and *P*=0.04 in the *Z* test). The results of EC, GC, and LC did not have heterogeneity (EC: Chi^2^ = 1.02, *P*=0.60, and *I*^2^ = 0%; GC: Chi^2^ = 1.96, *P*=0.58, and *I*^2^ = 0%; LC: Chi^2^ = 1.79, *P*=0.77, and *I*^2^ = 0%), but there was substantial heterogeneity among trials for CRC (Chi^2^ = 4.67, *P*=0.10, and *I*^2^ = 57%) (Figure 3(a)).

TSA suggested that the accrued information size (*n* = 1137) was 52% of RIS (*n* = 2176), while the cumulative Z-curve crossed the trial sequential monitoring boundary, indicating that current evidence was sufficient to reach a firm conclusion (Figure 3(b)). In addition, the GRADE assessment revealed that the quality of the evidence in ORR was moderate (Table 2).

#### 3.2.2. DCR

**Figure 4:**
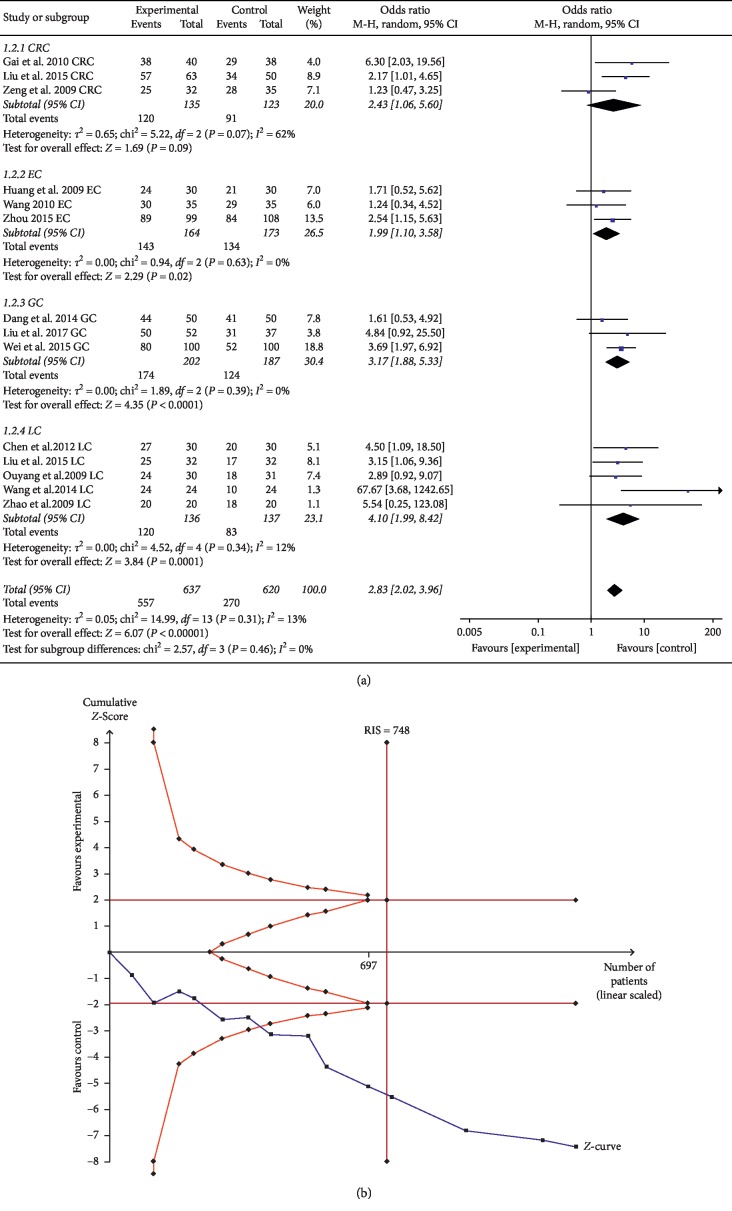
(a) Forest plot of DCR in the experimental group and control group. (b) Trial sequential analysis of DCR.

The DCR of ginsenoside Rg3 combined with chemotherapy was evaluated in a total of 14 trials comprising 1,257 patients. The results suggested that ginsenoside Rg3 combined with chemotherapy could statistically significantly enhance the DCR of EC, GC, and LC compared with chemotherapy alone (for EC, OR = 1.99, 95% CI: 1.10–3.58, and *P*=0.02 in the *Z* test; for GC, OR = 3.17, 95% CI: 1.88–5.33, and *P* < 0.0001 in the *Z* test; for LC, OR = 4.10, 95% CI: 1.99–8.42, and *P*=0.0001 in the *Z* test), and none of the three studies showed obvious heterogeneity (for EC, Chi^2^ = 0.94, *P*=0.63, and *I*^2^ = 0%; for GC, Chi^2^ = 1.89, *P*=0.39, and *I*^2^ = 0%; for LC, Chi^2^ = 4.52, *P*=0.34, and *I*^2^ = 12%). However, there was no statistically significant difference in DCR for CRC (OR = 2.74, 95% CI: 0.85–8.79, and *P*=0.09 in the *Z* test) (Figure 4(a)).

TSA showed that the accrued information size (*n* = 1257) was more than that of RIS (*n* = 748), and the cumulative Z-curve crossed the trial sequential monitoring boundary, suggesting that the trials sufficiently drew reliable conclusions (Figure 4(b)). In addition, the GRADE assessment revealed that the quality of the evidence in DCR was moderate (Table 2).

#### 3.2.3. SR

**Figure 5:**
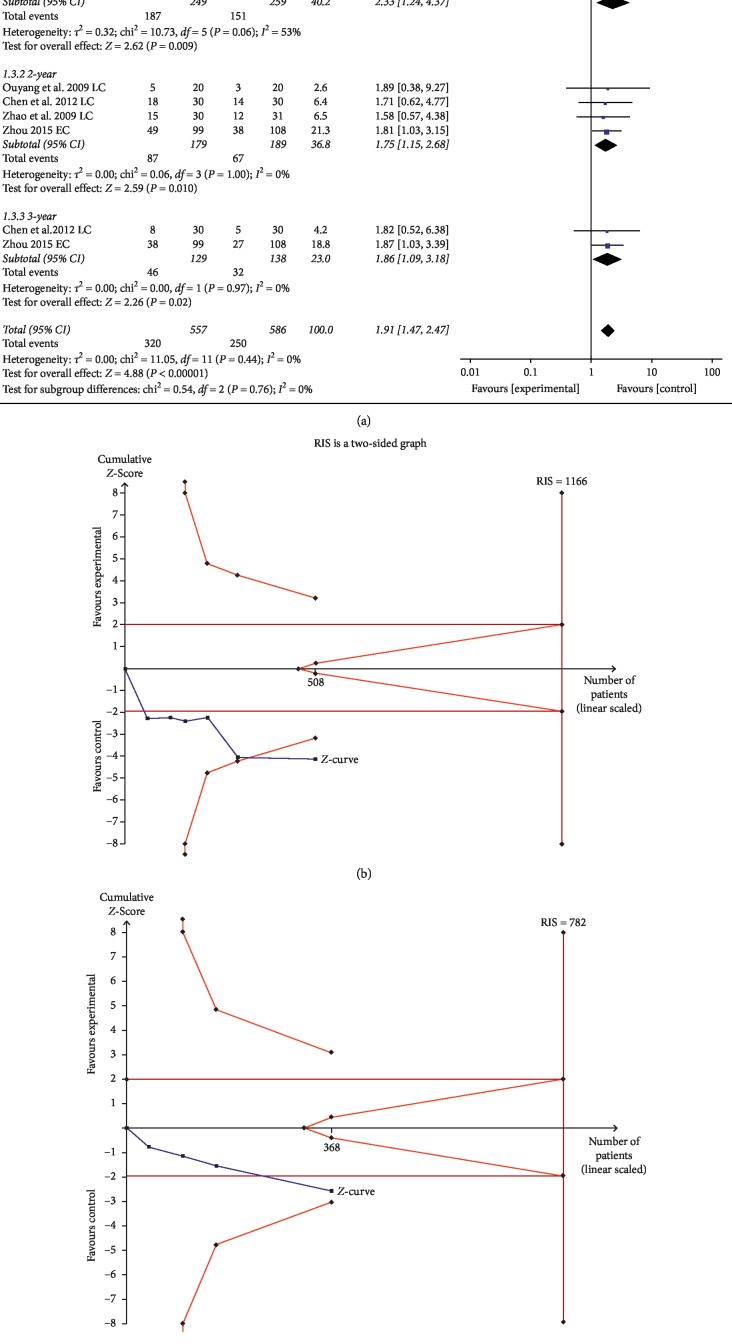
(a) Forest plot of SR in the experimental group and control group. Trial sequential analysis of (b) 1-year SR and (c) 2-year SR.

The one-year SR between the experimental group and control group was reported in 6 trials comprising 508 patients. Compared with chemotherapy alone, the 1-year SR was significantly improved after using ginsenoside Rg3 combined with chemotherapy (OR = 2.33, 95% CI = 1.24–4.37, *P*=0.009), while the data had substantial heterogeneity with Chi^2^ = 10.73, *P*=0.06, *I*^2^ = 53%. Four trials comprising 368 patients focused on 2-year SR between the experimental and control groups; the pooled data showed that ginsenoside Rg3 combined with chemotherapy was significantly better at increasing patients' 2-year SR (OR = 1.75, 95% CI: 1.15–2.68, *P*=0.01) without heterogeneity (Chi^2^ = 0.06, *P*=1.00, *I*^2^ = 0%). In addition, two trials comprising 267 patients reporting 3-year SR indicated that ginsenoside Rg3 combined chemotherapy can increase the 3-year SR (OR = 1.86, 95% CI: 1.09–3.18, *P*=0.02), and the pooled data did not show heterogeneity (Chi^2^ = 0.00, *P*=0.97, *I*^2^ = 0%) (Figure 5(a)).

However, TSA showed that the accrued information size in 1-year SR (*n* = 508) was 44% of RIS (*n* = 1166) and 2-year SR (*n* = 368) was 47% of RIS (*n* = 782). The cumulative Z-curve of 1-year SR crossed the trial sequential monitoring boundary, indicating that current evidence was sufficient to reach a firm conclusion (Figure 5(b)), but the cumulative Z-curve of 2-year SR did not cross the trial sequential monitoring boundary, indicating that current evidence was not sufficient to reach a firm conclusion (Figure 5(c)). In addition, the GRADE assessment revealed that the quality of the evidence in SR was moderate (Table 2).

#### 3.2.4. KPS

**Figure 6:**
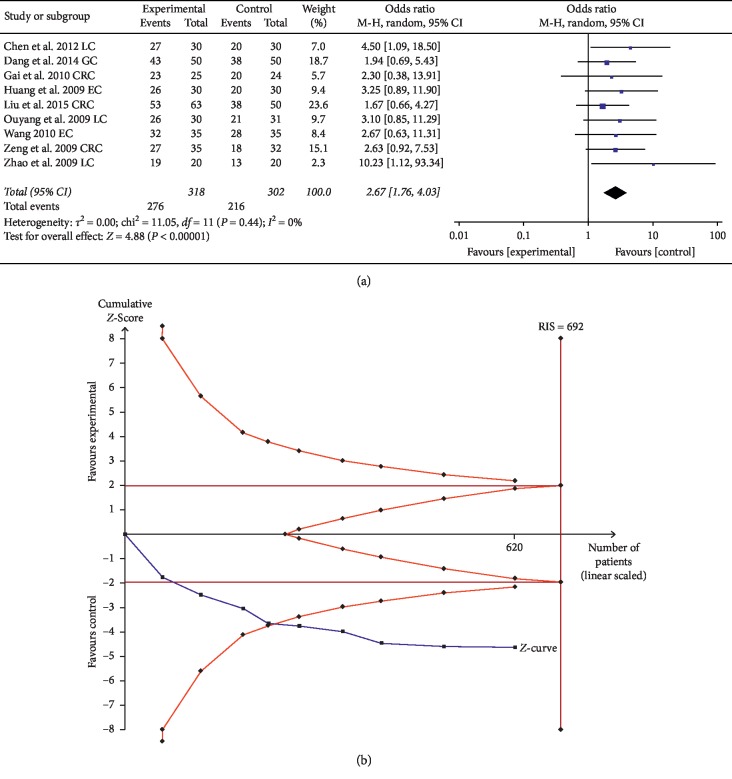
(a) Forest plot for the evaluation of KPS in the experimental group and control group. (b) Trial sequential analysis of KPS.

Nine trials comprising 620 patients evaluated the improvement of KPS using ginsenoside Rg3 combined with chemotherapy. The results suggested that KPS in the experimental group was significantly higher than that of control group (the OR = 2.67, 95% CI: 1.76–4.03, and *P* < 0.00001 in the *Z* test), and the data did not indicate heterogeneity with Chi^2^ = 3.43, *P*=0.90, and *I*^2^ = 0% (Figure 6(a)).

TSA showed that the accrued information size (*n* = 620) was 90% of RIS (*n* = 692), and the cumulative Z-curve crossed the trial sequential monitoring boundary, indicating that the evidence will reach a firm conclusion when the sample size is sufficient (Figure 6(b)). In addition, the GRADE assessment revealed that the quality of the evidence in KPS was moderate (Table 2).

### 3.3. Secondary Outcomes

#### 3.3.1. Gastrointestinal Dysfunction

**Figure 7:**
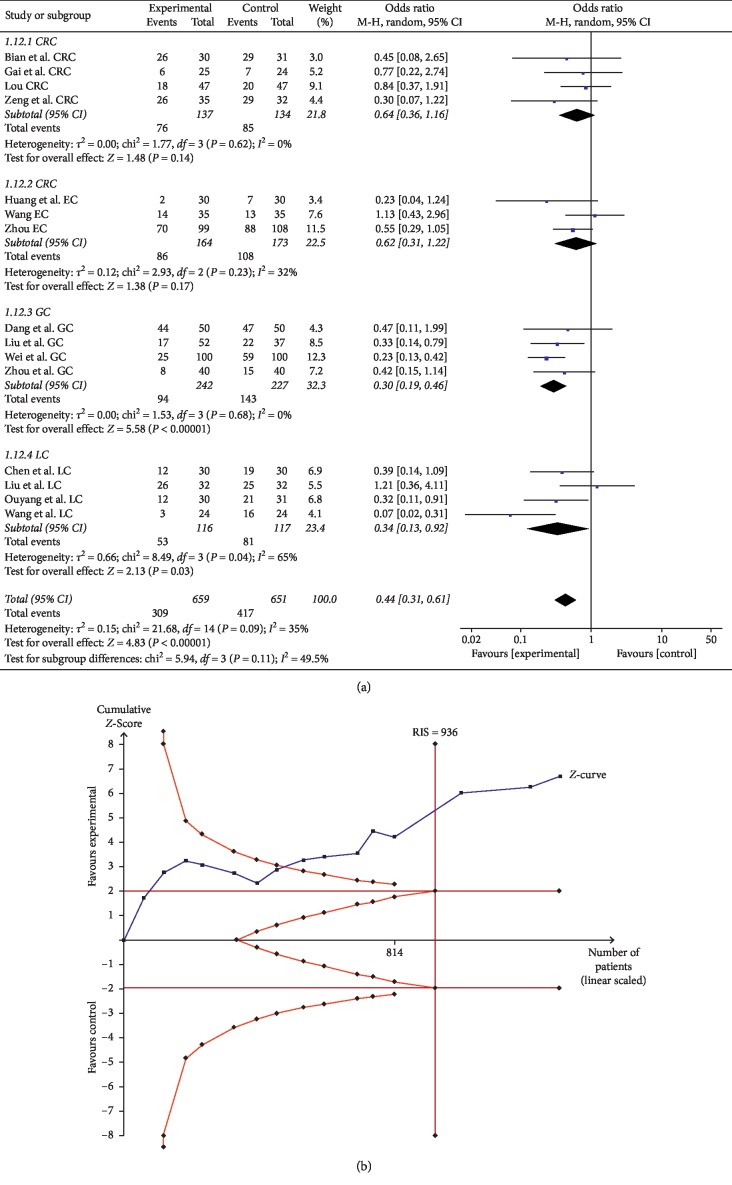
(a) Forest plot for evaluating the gastrointestinal dysfunction in the experimental group and control group. (b) Trial sequential analysis of the gastrointestinal dysfunction.

Fifteen trials comprising 1,310 patients evaluated the gastrointestinal dysfunction of digestive system cancer, including nausea, vomiting, constipation, and diarrhea. There were four trials each related to CRC, GC, and LC and three trials related to EC. Compared with the control group, the gastrointestinal dysfunction in the experimental group was significantly lower in GC and LC (for GC, OR = 0.30, 95% CI: 0.19–0.46, and *P* < 0.00001 in the *Z* test; for LC, OR = 0.34, 95% CI: 0.13–0.92, and *P*=0.03 in the *Z* test). Heterogeneity was not observed in GC (Chi^2^ = 1.53, *P*=0.68, and *I*^2^ = 0%), but was obvious in LC (Chi^2^ = 8.49, *P*=0.04, and *I*^2^ = 65%). As for CRC and EC, there was no statistically significant difference between the control and experimental groups (for CRC, OR = 0.64, 95% CI: 0.36–1.16, and *P*=0.14 in the *Z* test; for EC, OR = 0.62, 95% CI: 0.31–1.22, and *P*=0.17 in the *Z* test) (Figure 7(a)).

TSA showed that the accrued information size (*n* = 1310) was more than that of RIS (*n* = 936), and the cumulative Z-curve crossed the trial sequential monitoring boundary, indicating that the trials sufficiently drew reliable conclusions (Figure 7(b)). In addition, the GRADE assessment revealed that the quality of the evidence in gastrointestinal dysfunction was moderate (Table 2).

#### 3.3.2. The Decline of Leucocyte Count

**Figure 8:**
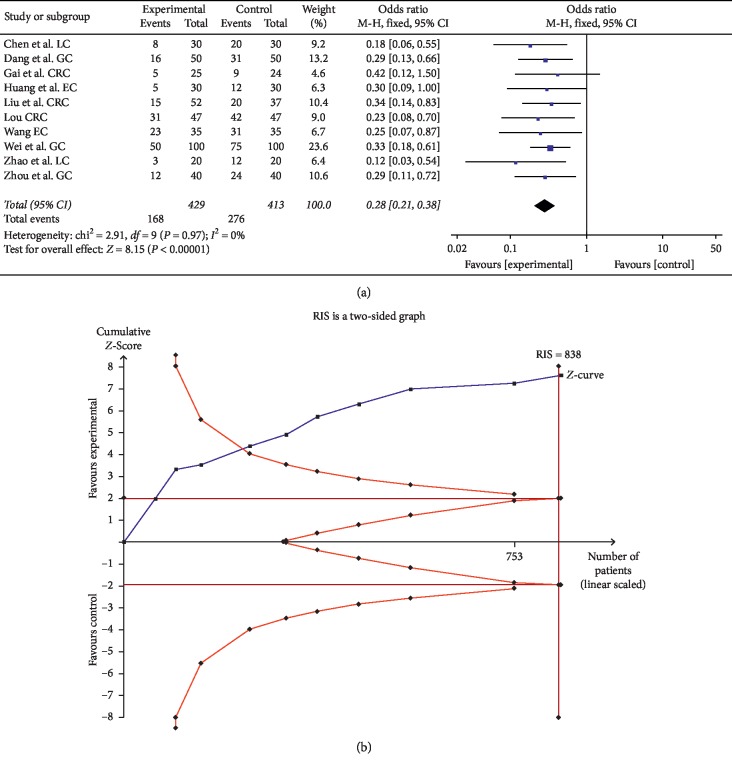
(a) Forest plot for evaluating the decline of leucocyte count in the experimental group and control group. (b) Trial sequential analysis of the decline of leucocyte count.

Ten trials comprising 842 patients compared the decline of leukocyte count. The decline in the experimental group was significantly lower than in the control group (OR = 0.28, 95% CI: 0.21–0.38, and *P* < 0.00001 in the *Z* test), and the data among studies did not show heterogeneity (Chi^2^ = 3.26, *P*=0.97, and *I*^2^ = 0%) (Figure 8(a)).

TSA showed that the accrued information size (*n* = 842) was more than that of RIS (*n* = 838), and the cumulative Z-curve crossed the trial sequential monitoring boundary, indicating that the evidence will reach a firm conclusion when the sample size is sufficient (Figure 8(b)). In addition, the GRADE assessment revealed that the quality of the evidence in the leucocyte count was moderate (Table 2).

#### 3.3.3. Hepatic and Renal Dysfunction

**Figure 9:**
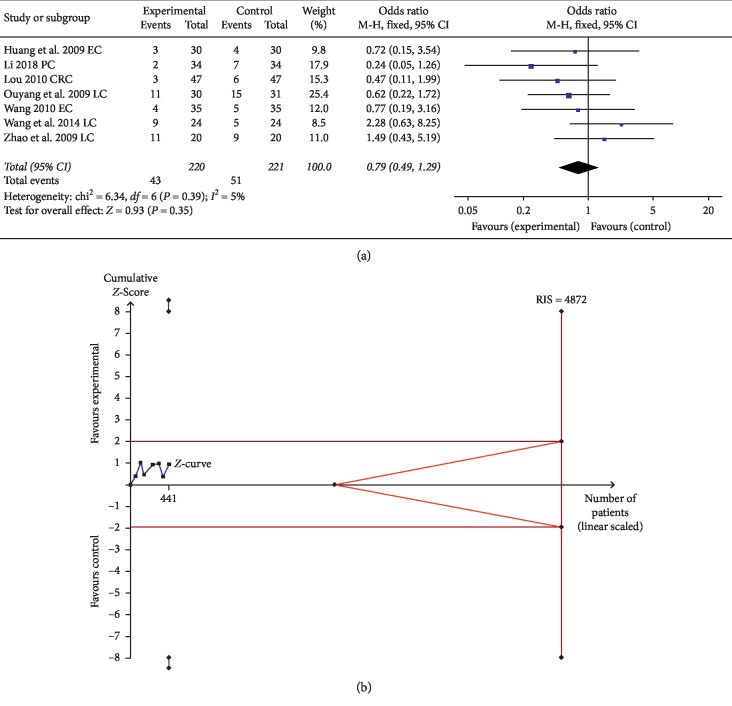
(a) Forest plot for evaluating the hepatic and renal dysfunction in the experimental group and control group (b) Trial sequential analysis of the hepatic and renal dysfunction.

Seven trials comprising 441 patients evaluated hepatic and renal dysfunction of digestive system cancer after treatment. The data did not reveal a statistically significant difference between the control and experimental groups (OR = 0.79, 95% CI: 0.49 to 1.29, and *P*=0.35 in the *Z* test) (Figure 9(a)).

TSA suggested that the accrued information size (*n* = 441) was 9% of RIS (*n* = 4872), and the cumulative Z-curve did not cross the trial sequential monitoring boundary, indicating that the trials were not sufficient to reach a firm conclusion (Figure 9(b)). In addition, the GRADE assessment revealed that the quality of the evidence in the hepatic and renal dysfunction was low (Table 2).

### 3.4. Sensitivity Analysis

The ORR of EC, GC, and LC, the DCR of EC and GC, the 2-year and 3-year SR, KPS, gastrointestinal dysfunction of GC, and the decline of leucocyte count were improved significantly without heterogeneity. The DCR of LC had a low heterogeneity, whereas the ORR of CRC and the 1-year SR and gastrointestinal dysfunction of LC indicated a substantial heterogeneity. The DCR of CRC and hepatic and renal dysfunction and gastrointestinal dysfunction of CRC and EC were not statistically significant.

The heterogeneity of the included studies were evaluated by the Chi^2^ and *I*^2^ test, with *P* < 0.10 or *I*^2^ > 50% defined as indicating heterogeneity. As shown in Table 3, there was significant heterogeneity in the ORR of CRC (*I*^2^ = 57%, *P*=0.10), DCR of CRC (*I*^2^ = 62%, *P*=0.07), 1-year SR (*I*^2^ = 53%, *P*=0.06), and gastrointestinal dysfunction of LC (*I*^2^ = 65%, *P*=0.04). In addition to these four evaluation data, other studies had low or no heterogeneity. As shown in Table 4, for the ORR of CRC, after excluding the study Gai et al., the heterogeneity was not observed (*I*^2^ = 0%, *P*=0.37), but the data was not statistically significant. For the DCR of CRC, after excluding the study Zeng et al., there was no heterogeneity in the result (*I*^2^ = 0%, *P*=0.78). For the 1-year SR, after excluding the study Zhou et al., the data had no heterogeneity (*I*^2^ = 0%, *P*=0.70). For gastrointestinal dysfunction of LC, after excluding the study Liu et al., the heterogeneity was significantly reduced (*I*^2^ = 45%, *P*=0.16). Therefore, the data of this study generally had good consistency.

### 3.5. Publication Bias

**Figure 10:**
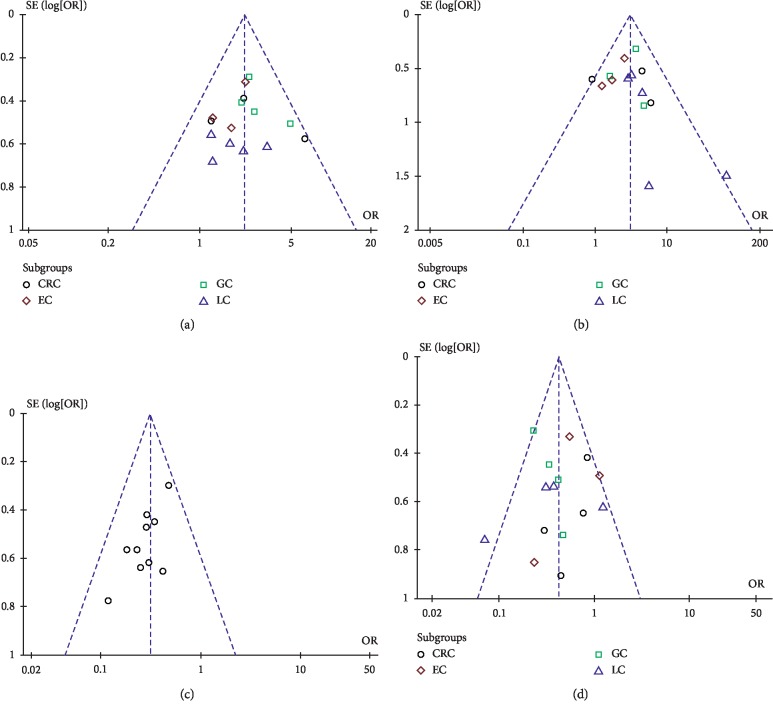
The publication bias analysis: (a) 10A, ORR; (b) 10B, DCR; (c) 10C, the decline of leucocyte count; (d) 10D, gastrointestinal dysfunction.

The funnel plots were nearly symmetrical in ORR (Figure 10(a)), DCR (Figure 10(b)), and gastrointestinal dysfunction (Figure 10(d)), so these three studies were objectively reported and did not have publication bias. The funnel plot was asymmetrical in the decline of leucocyte count (Figure 10(c)). Therefore, we further used Egger's test to evaluate their publication bias. As shown in Table 5, the ORR, DCR, and gastrointestinal dysfunction had a *P* value of *P* > 0.05, so Egger's publication test suggested that there was no publication bias in these three studies, but Egger's publication test suggested that the decline of leucocyte count had a risk of publication bias, with *P* < 0.05.

## 4. Discussion

### 4.1. Summary of Previous Evidence

Chemotherapy is currently the most common treatment for digestive system cancers, but its many side effects may compromise the therapeutic effect. In TCM, ginseng can reinforce vital energy, which is consistent with enhancement of resistance in modern medicine. Many randomized, prospective clinical trials indicate that ginsenoside Rg3 can effectively treat digestive system cancer and improve its side effects. For example, Tang et al. [33, 34] reported that ginsenoside Rg3 can target cancer stem cells and tumor angiogenesis to inhibit CRC progression by downregulating C/EBPβ/NF-κB signaling. Aziz et al. [35] found that ginsenoside Rg3 induces FUT4-mediated apoptosis in *H. pylori* CagA-treated GC cells by regulating SP1 and HSF1 expressions. Ge et al. [36] confirmed that ginsenoside Rg3 enhances radiosensitization of hypoxic EC cell lines through VEGF and hypoxia inducible factor-1α. Finally, Sun et al. [37] confirmed that ginsenoside Rg3 inhibits the migration and invasion of LC cells by increasing the protein expression of ARHGAP9. Therefore, ginsenoside Rg3 may serve well as a therapeutic treatment for digestive system cancers.

### 4.2. Summary of Our Evidence

In order to evaluate the efficacy and safety of ginsenoside Rg3 combined with chemotherapy for digestive system cancer accurately, we completed the meta-analysis and systematic review comprehensively for the first time and reported 18 trials with a total of 1,531 patients applied RCTs. In the quality assessment, all the included trials were RCTs with a low risk in attrition bias and reporting bias. About one-third of the trials had a low risk in performance bias and detection bias, two-thirds of the studies had a low risk in other bias. In the publication bias, the funnel plots and Egger's test showed that there was no publication bias in the results of ORR, DCR, and gastrointestinal dysfunction, but the decline of leucocyte count had publication bias. In the sensitivity analysis, after excluding some under- or overestimated trials, the data of this study generally have good consistency. TSA revealed that the trials were of sufficient standard to draw reliable conclusions that ginsenoside Rg3 combined with chemotherapy could improve the ORR, DCR, 1-year SR, KPS, gastrointestinal dysfunction, and the decline of *leucocyte* count. In addition, the GRADE assessment showed that the quality of evidences were moderate. Therefore, according to the research evidence we had included, ginsenoside Rg3 combined with chemotherapy can significantly control disease progression and reduce the side effects caused by chemotherapy in digestive system cancer.

### 4.3. The Limitation and Expectation

This study has three limitations. First, there is a geographical bias because ginsenoside Rg3 is used for cancer treatment mostly in China. Performing trials in other countries may confirm that ginsenoside Rg3 can be used effectively with chemotherapy for digestive system cancers. Second, some of the included studies were unclear in the allocation concealment and blinding method, although this is unlikely to have a serious impact on the assessment due to the objective criteria (WHO and RECIST). Third, it is not clear which chemotherapy regimens combined with ginsenoside Rg3 is most effective. With the continuous supplementation and improvement of RCTs in this field, we can explore the best chemotherapy regimen combined with ginsenoside Rg3 for digestive system cancer.

## 5. Conclusion

Despite some limitations, this meta-analysis and systematic review provides assurance that ginsenoside Rg3 combined with chemotherapy can enhance therapeutic effect, improve quality of life, and alleviate side effects of chemotherapy for digestive system cancer patients. In future, we will conduct large and well-designed RCTs to test the above conclusions and explore the best chemotherapy regimen combined with ginsenoside Rg3 for digestive system cancer.

## Figures and Tables

**Table 1:** Characteristics of the included studies.

Reference	Year	Disease	Sample size (E/C)	Sex (M/F)		Age(years) (E/C)	Interventions		Follow-up time	Outcome measures	Criteria for efficacy
				E	C		E	C			
Dang et al. [15]	2014	GC (advanced)	100(50/50)	Unknown		61.06 ± 4.21/60.21 ± 4.62	Rg3 +C	DDP + placebo	8 months	①②④⑤	WHO
Liu et al. [16]	2017	GC (IIIA: 15, IIIB: 52, IIIC: 22)	89(52/37)	29/23	20/17	45.1 ± 8.5/44.8 ± 9.3	Rg3 +C	FOLFOX4	6 months	①②⑤	RECIST
Wei et al. [17]	2015	GC (III-IV)	200(100/100)	68/32	70/30	18–75(48.5 ± 6.7)/19–74(46.5 ± 5.7)	Rg3 +C	Tegafur + DDP	5 years	①②⑤	RECIST
Zhou et al. [18]	2013	GC (advanced)	80(40/40)	Unknown		44–73(61)/43–72 (60)(median age)	Rg3 +C	PTX	4 years	①③⑤	WHO
Bian et al. [19]	2014	CRC (advanced)	61(30/31)	18/12	17/14	54.6 ± 9.6/57.1 ± 7.4	Rg3 +C	XELOX	2 years	⑤	RECIST
Gai et al. [20]	2010	CRC (advanced)	49(25/24)	28/21		35–75	Rg3 +C	XELOX	9 weeks	①②④⑤	WHO
Liu et al. [21]	2015	CRC III (77%), IV (23%)	113(63/50)	53/60		18–80 (median age 55)	Rg3 +C	Fluorouracil + placebo	14 months	①②④⑤	WHO
Lou [22]	2010	CRC (II-III)	94(47/47)	Unknown		41–75	Rg3 +C	FOLFOX6	2 months	⑤	RECIST
Zeng et al. [23]	2009	CRC (IV)	67(35/32)	25/10	22/11	51–69	Rg3 +C	FOLFOX4	2 months	①②④⑤	WHO
Huang et al. [24]	2009	EC (IV)	60(30/30)	17/13	18/12	45–75(54)/42–74 (52)(median age)	Rg3 +C	GP(GEM + DDP)	1 year	①②③④⑤	WHO
Wang [25]	2010	EC (advanced)	70(35/35)	46/24		32–78 (median age 56)	Rg3 +C	NP(NVB + DDP)	6 weeks	①②④⑤	WHO
Zhou [26]	2015	EC (T_3_-T_4a_)	207(99/108)	65/34	70/38	48–76(61.6 ± 7.6)/50–79(63.5 ± 8.4) (average age)	Rg3 +C	TP(PTX + DDP)	3 years	①②③⑤	WHO
Liu et al. [27]	2015	LC (advanced)	64(32/32)	18/14	17/15	52.13 ± 10.25/53.27 ± 11.59	Rg3 +C	TACE + lipiodol + pirarubicin	3 months	①②⑤	GPCRNCM
Ouyang et al. [28]	2009	LC (II: 9, III: 39, IV: 13)	61(30/31)	25/5	23/8	21–72 (median age 50)	Rg3 +C	TACE + lipiodol	2 years	①②③④⑤	GPCRNCM
Zhao et al. [29]	2009	LC (advanced)	40(20/20)	14/6	16/4	59.5 ± 11.6/62.3 ± 10.8	Rg3 +C	TACE + GP + ML	2 years	①②③④⑤	GPCRNCM
Chen et al. [30]	2012	LC (advanced)	60(30/30)	Unknown		Unknown	Rg3 +C	TACE + Fluorouracil + DDP + ML	3 years	②③④⑤	WHO
Wang et al. [31]	2014	LC (advanced)	48(24/24)	29/19		52.13 ± 3.65	Rg3 +C	TACE + mitomycin + adriamycin+	6 months	①②⑤	Unknown
Li [32]	2018	PC (III: 59, IV: 9)	68(34/34)	21/13	20/14	52.2 ± 3.8/52.3 ± 3.9	Rg3 +C	GEM	4 months	⑤	Unknown

*Note.* E: experimental group; C: control group; Rg3: Rg3 20 mg po.Bid; PTX: paclitaxel; DDP: cisplatin; XELOX: oxaliplatin + capecitabine; FOLFOX4/FOLFOX6: oxaliplatin + calcium folinate + fluorouracil; GP (GEM + DDP): gemcitabine + cisplatin; TP (PTX + DDP): paclitaxel + cisplatin; NP (NVB + DDP): vinorelbine + cisplatin; TACE: transcatheter arterial chemoembolization; ML: mitomycin + lipiodol; WHO: World Health Organization; RECIST: the response evaluation criteria in solid tumors; GPCRNCM: Guiding Principles for Clinical Research of New Chinese Medicines; ① ORR; ② DCR; ③ SR; ④ KPS; ⑤ side effects.

**Table 2:** GRADE evidence profile of clinical efficacy.

Studies (follow-up time)	Quality assessment					Quality	Importance
	Risk of bias	Inconsistency	Indirectness	Imprecision	Other considerations		
ORR (4–16 weeks)	Serious^1^	No serious inconsistency	No serious indirectness	No serious imprecision	None	⊕⊕⊕Ο	Critical
						MODERATE	

DCR (4–16 weeks)	Serious^1^	No serious inconsistency	No serious indirectness	No serious imprecision	None	⊕⊕⊕Ο	Critical
						MODERATE	

SR (1–3 years)	Serious^1^	No serious inconsistency	No serious indirectness	No serious imprecision	None	⊕⊕⊕Ο	Critical
						MODERATE	

KPS (4–16 weeks)	Serious^1^	No serious inconsistency	No serious indirectness	No serious imprecision	None	⊕⊕⊕Ο	Critical
						MODERATE	

The decline of leucocyte count (4–8 weeks)	Serious^1^	No serious inconsistency	No serious indirectness	No serious imprecision	None	⊕⊕⊕Ο	Important
						MODERATE	

Gastrointestinal dysfunction (4–16 weeks)	Serious^1^	No serious inconsistency	No serious indirectness	No serious imprecision	None	⊕⊕⊕Ο	Important
						MODERATE	

The hepatic and renal dysfunction (4–16 weeks)	Serious^1^	Serious^2^	No serious indirectness	No serious imprecision	None	⊕⊕ΟΟ	Important
						LOW	

^1^Most domains had unclear methodological bias risk; ^2^the included studies had inconsistent result, and therefore the evidence was rated down by one level.

**Table 3:** Sensitivity analysis.

Analysis		Sensitivity analysis	*I* ^2^	Cochran P	OR (95% CI)	*P*
ORR	CRC	REM	57%	*P*=0.10	2.43 [1.06, 5.60]	*P*=0.04
	EC		0%	*P*=0.60	1.85 [1.17, 2.92]	*P*=0.009
	GC		0%	*P*=0.58	2.60 [1.78, 3.80]	*P* < 0.00001
	LC		0%	*P*=0.77	1.77 [1.04, 3.03]	*P*=0.04

DCR	CRC	REM	62%	*P*=0.07	2.74 [0.85, 8.79]	*P*=0.09
	EC		0%	*P*=0.63	1.99 [1.10, 3.58]	*P*=0.02
	GC		0%	*P*=0.39	3.17 [1.88, 5.33]	*P* < 0.0001
	LC		12%	*P*=0.34	4.10 [1.99, 8.42]	*P*=0.0001

SR	1-year	REM	53%	*P*=0.06	2.33 [1.24, 4.73]	*P*=0.009
	2-year		0%	*P*=1.00	1.75 [1.15, 2.68]	*P*=0.01
	3-year		0%	*P*=0.97	1.86 [1.09, 3.18]	*P*=0.02

KPS		FEM	0%	*P*=0.90	2.67 [1.76, 4.03]	*P* < 0.00001

Gastrointestinal dysfunction	CRC	REM	0%	*P*=0.62	0.64 [0.36, 1.16]	*P*=0.14
	EC		32%	*P*=0.23	0.62 [0.31, 1.22]	*P*=0.17
	GC		0%	*P*=0.68	0.30 [0.19, 0.46]	*P* < 0.00001
	LC		65%	*P*=0.04	0.34 [0.13, 0.92]	*P*=0.03

The decline of leucocyte count		FEM	0%	*P*=0.97	0.28 [0.21, 0.38]	*P* < 0.00001

Hepatic and renal dysfunction		FEM	5%	*P*=0.39	0.79 [0.49, 1.29]	*P*=0.35

**Table 4:** Sensitivity analysis via excluding the under- or overestimated trials.

Indicators		Trials	OR (95% CI)	*I* ^2^	Excluded studies [Reference]	Trials	OR (95% CI)	*I* ^2^
ORR (CRC)		3	2.43[1.06, 5.60]	57%	Zeng et al. CRC [23]	2	3.39[1.21, 9.54]	57%
DCR (CRC)		3	2.74[0.85, 8.79]	62%	Zeng et al. CRC [23]	2	4.85[2.04, 11.53]	0%
SR (1-year)		6	2.33[1.24, 4.37]	53%	Zhou et al. GC [18]	5	1.77 [1.16, 2.70]	0%
Gastrointestinal dysfunction	CRC	4	0.64 [0.36, 1.16]	0%	No statistical significance			
	EC	3	0.62 [0.31, 1.22]	32%	Wang EC [25]	2	0.49 [0.27, 0.90]	0%
	LC	4	0.34[0.13, 0.92]	65%	Liu et al. LC [16]	3	0.24 [0.09, 0.58]	45%
Hepatic and renal dysfunction		7	0.79 [0.49, 1.29]	5%	Wang et al. LC [31]	5	0.54 [0.30, 0.99]	0%
					Zhao et al. LC [29]			

**Table 5:** Egger's publication test.

Detection indicators	*P* value
ORR	0.804
DCR	0.394
The decline of leucocyte count	0.009
Gastrointestinal dysfunction	0.549
